# A Current Update on Human Papillomavirus-Associated Head and Neck Cancers

**DOI:** 10.3390/v11100922

**Published:** 2019-10-09

**Authors:** Ebenezer Tumban

**Affiliations:** Department of Biological Sciences, Michigan Technological University, 1400 Townsend Dr, Houghton, MI 49931, USA; etumban@mtu.edu; Tel.: +1-906-487-2256; Fax: +1-906-487-3167

**Keywords:** HPV, oral transmission, head and neck cancers, HPV vaccines, HIV and AIDS, head and neck cancer treatment

## Abstract

Human papillomavirus (HPV) infection is the cause of a growing percentage of head and neck cancers (HNC); primarily, a subset of oral squamous cell carcinoma, oropharyngeal squamous cell carcinoma, and laryngeal squamous cell carcinoma. The majority of HPV-associated head and neck cancers (HPV + HNC) are caused by HPV16; additionally, co-factors such as smoking and immunosuppression contribute to the progression of HPV + HNC by interfering with tumor suppressor miRNA and impairing mediators of the immune system. This review summarizes current studies on HPV + HNC, ranging from potential modes of oral transmission of HPV (sexual, self-inoculation, vertical and horizontal transmissions), discrepancy in the distribution of HPV + HNC between anatomical sites in the head and neck region, and to studies showing that HPV vaccines have the potential to protect against oral HPV infection (especially against the HPV types included in the vaccines). The review concludes with a discussion of major challenges in the field and prospects for the future: challenges in diagnosing HPV + HNC at early stages of the disease, measures to reduce discrepancy in the prevalence of HPV + HNC cases between anatomical sites, and suggestions to assess whether fomites/breast milk can transmit HPV to the oral cavity.

## 1. Introduction

Head and neck cancers (HNC) account for ~4.8% of cancers and they are associated with a similar percentage of cancer mortality worldwide [1]. Ninety percent of HNC arises from squamous epithelial cells lining the oral cavity, pharynx, larynx, or, more rarely, the nasal cavity. These include: (i) oral squamous cell carcinomas (OSCC), which are cancers that arise from lips, tongue, floor of the mouth, oral cavity, etc.; (ii) oropharyngeal squamous cell carcinomas (OPSCC), which are cancers that arise from the base of the tongue, the soft palate, tonsils, back of the throat; (iii) laryngeal squamous cell carcinomas (LSCC), which arise from the supraglottis, glottis, subglottis; (iv) nasal squamous cell carcinomas (NSCC; to a lesser extent), which arise from squamous epithelial cells lining the nasal cavity and paranasal sinuses [2,3,4]. The remaining 10% of HNC arises from lymphocytes, cells of connective tissue (muscle, blood vessel), and cells of the salivary glands [3]. Many factors/co-factors have been linked to HNC: alcohol consumption [5,6], smoking, and/or chewing of tobacco [7,8] increase the risk for HNC. Alcohol consumption is associated with ~5% of HNC cases, tobacco use is associated with ~34%, while consumption of alcohol in combination with tobacco use is associated with 36% of ~HNC cases [5,7,9]; thus, ~75% of HNC (i.e., squamous cell carcinomas) are caused by alcohol and tobacco use. The remaining percentage (~25%; worldwide average) of HNC cases is caused by human papillomaviruses (HPVs) [10,11]. HPVs also cause almost all cases of cervical cancer, a percentage of other anogenital cancers (vaginal, vulva, anal, penile, etc.), and almost all cases of genital warts [12]. This review focuses only on HPV-associated HNC (HPV + HNC): it summarizes the transmission of HPV to the head and neck region, the contribution of HPV to HNC (specifically OSCC, OPSCC, and LSCC). It also highlights measures currently being used (with potentials) to protect against oral transmission of HPVs as well as challenges facing the diagnosis and treatment of HPV + HNC.

## 2. HPV, Genome, and Oncogenes

**Figure 1:**
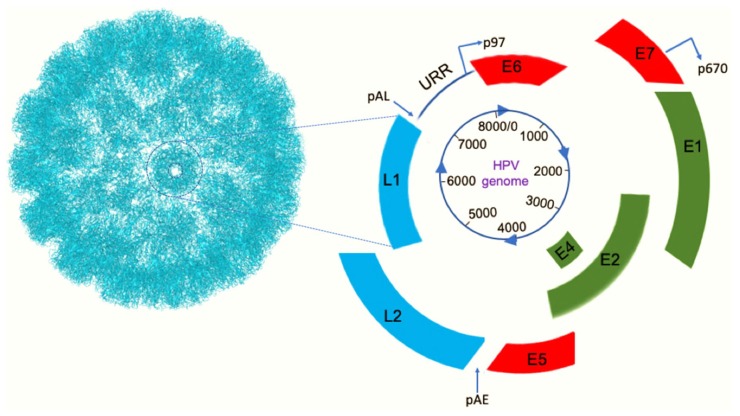
A schematic of the genome of HPV16 and capsid proteins. Right image: genes that code for early proteins (E1, E2, E4, E5, E6, and E7), are shown in red and green colors; E5, E6, and E7 are oncogenes. Genes that code for late proteins (L1 and L2; capsid proteins) are shown in light blue color. URR (upstream regulatory region) contains origin of replication, enhancer elements, and early promoter (p97); URR controls viral replication. p670 is the late promoter. pAE and pAL are early polyadenylation and late polyadenylation sites, respectively. Left image: The L1 protein forms pentamers (one is circled) and each pentamer has an L2 protein at its center (not shown). Seventy-two copies of the pentamers assemble to form an icosahedral capsid.

More than 220 HPV types have been identified as of 2019 [13]. HPVs are non-enveloped double-stranded DNA viruses with a circular genome of ~8000 base-pairs; HPVs infect and replicate in epithelial cells in the skin and mucosal regions [14]. Its genome codes for six nonstructural genes (early genes: E1, E2, E4, E5, E6, and E7) and two structural genes (late genes: L1 and L2) (Figure 1). L1 and L2 are required for viral assembly while E1, E2, and E4 are required for viral replication and regulation of replication; E5, E6, and E7 on the other hand are oncogenes and are implicated in HPV-associated cellular transformation [14,15]. Following HPV infection, the genome is maintained as an episome in the nucleus; integration of E6 and E7 into the host chromosome and persistent expression of these oncogenes interferes with the functioning of cell cycle regulator (tumor suppressor) proteins. E6 binds to p53 via E6AP (also known as ubiquitin-protein ligase E3A; UBE3A) and targets p53 for degradation via a proteasome-mediated pathway [16]. E6 and to some extent E7 have also been reported to enhance telomerase activity especially its catalytic unit, human telomerase reverse transcriptase (hTERT; elongates telomeres); reviewed in ref. [17]. Under normal circumstances, hTERT is constitutively repressed in somatic cells. However, E6 removes transcriptional suppressors from the promoter of hTERT and hypomethylates and acetylates the promoter; reviewed in ref. [17]. These changes cause the expression of hTERT, which then elongates telomeres (via replication) thus preventing end-to-end fusion of chromosomes (crisis) and apoptosis [18]; this provides an opportunity for the cell to continue to divide and become immortal. E7, on the other hand, binds to pRb protein, dissociating it from E2F (a transcription factor), and thus E2F drives the cell to enter, uncontrollably, the S phase of the cell cycle [19]. The inability of these cell cycle regulators to control cell division leads to uncontrolled cell proliferation and ultimately cancer [20]. In addition to p53 and pRb inactivation, other cancer-related pathway proteins (independent of p53 and pRb), especially those involved in regulating the cell cycle, are also targeted by E6 and E7 oncoproteins. For example, E7 binds and inactivates cell-cycle regulatory proteins such as inhibitors of the cell cycle (cyclin-depend kinase inhibitors: p21^CIP1^ and p27^Kip1^) while at the same time, it binds to cyclin-dependent kinase 2 and enhances its activity [20,21,22]; both events promote cell division. Furthermore, E6 and E7 oncoproteins also target, indirectly, non-coding RNAs (known as microRNAs; miRNA) associated with tumor progression/suppression. For example, E6 and E7 increase the levels of miRNA-21 (oncogenic miRNA), which negatively targets the expression of PTEN (a tumor suppressor gene) [23,24]. At the same time, E5, E6, and E7 expression in cervical cancer cell lines (CaSki and siHa) downregulate the expression of some microRNAs (tumor suppressive miRNAs: miRNA-22, miRNA-148a-3p, miRNA-190a-5p, miRNA-450, miRNA-455, and miRNA-203), which control/inhibit cell proliferation and as such, the oncoproteins promote cell proliferation [24,25,26]. Thus, HPV oncogenes enhance/decrease miRNA levels and the effect of the miRNAs on HPV-associated cancer depends on target genes (tumor suppressor genes versus proto-oncogenes).

It is also worth mentioning that E6 and E7 have other functions, in addition to serving as oncogenes, which help reprogram the cell to enhance the replication of HPV (e.g., antiviral responses). For example, E6 and E7 bind to interferon regulatory factor-3 protein and interferon regulatory factor-1 protein, respectively, and inactivate these proteins; this thus, inhibits interferon signaling pathways and allows the virus to evade the immune system [21]. In addition to these, E6 binds to death receptor (FADD) on the cells, thus blocking fas-mediated apoptosis [20,21,22]. This allows the cells to serve as a “factory” for the virus to continue to replicate.

E5 oncoprotein is believed to enhance the cellular transformation by activating epidermal growth factor receptors on epithelial cells [20,22].

HPVs are divided into two groups based on their association with neoplasia: low-risk types and high-risk types. Infections with low-risk HPV types (types 6, 11, 40–44, 54, 61, 72, 81, etc.) are associated with genital warts and recurrent respiratory papillomatosis (RRP) while infections with the high-risk HPV types (oncogenic types; types 16, 18, 26, 31, 33, 35, 39, 45, 51–53, 56, 58, 59, 66, 68, 70, 73, and 82) are associated with cancers (reviewed in ref. [12])

## 3. Transmission of HPVs to the Head and Neck Region

HPVs associated with HNC—unlike those associated with anogenital cancers and genital warts—are transmitted orally, primarily through oral sex; oral sex, defined in this review as contact of the oral region with the anogenital region such as vaginal, anal, penile, genital, etc. Studies have shown that oral sexual activities as well as an increase in the number of oral sexual partners increase oral transmission of HPV [27,28,29,30]. An increase in oral transmission of HPV ultimately leads to an increase in the infection of the head and neck region; a high number of HPV infections of the head and neck region has been reported in men compared to women [27,28,29,30]. This is probably due to a high number of men giving oral sex to HPV-infected partners. Thus, oral sex is associated with most cases of HPV infection of the head and neck region.

Deep (open-mouthed) kissing has also been reported to be associated with oral transmission of HPV. HPV has been detected in oral mucosal of men/women, without a history of oral sex, who had ≥10-lifetime deep kissing or had ≥5 deep kissing within a year [28,31]. Thus, deep kissing is associated with oral HPV transmission. Recently, other routes of nonsexual oral transmission have been reported or proposed.

### 3.1. Self-Inoculation or Autoinoculation

About 4% of patients with cervical HPV infection (HPV16, 35, or 45; associated with high-grade squamous intraepithelial lesion) have been reported to be co-infected with the same HPV types at the oral region [32,33]. Given the fact that HPV infection is localized and not systemic, it is likely that these HPV types were self-inoculated (in the absence of oral sex) from the genital region to the oral region. In fact, HPV has been detected in the fingernails and oral cavity of women with vaginal HPV infection [31,34]. This thus suggests that HPV can be transferred by self-inoculation from the genital region to the oral region through contaminated fingernails.

### 3.2. Vertical Transmission from Infected Mothers to Children

HPV has been detected in oral scrapings of ~32% of children born to mothers with genital HPV infection. More than half of the HPV-positive children were infected with the same HPV type detected in the mother’s genital region at the time of delivery (reviewed in ref. [35]). Other studies have documented that more than 30% of children born to mothers with genital HPV infection suffer from oral papilloma or RRP [35,36,37]; >90% of RRP is caused by HPV6 and HPV11 (these HPV types do not cause cancer). Thus, HPV can be transmitted from the genital region of a mother to the oral cavity of a child during birth. Another proposed method of mother to child transmission, which is still controversial, is by breast milk. HPV DNA (HPV6, 16, 18, 33, 45, 53, 56, 59, 66, and 82) has been detected in breast milk of lactating mothers [38,39]; however, HPV DNA types in the breast milk were different from those in children’s oral mucosal. For example, in one of these studies, when HPV16 DNA was detected (most detected type) in the breast milk of a lactating mother, a different HPV type (HPV6) was detected in the oral mucosal of the offspring and vice versa [38]. Given these results and others in the field, it is too early to tell whether HPV can be transmitted through breast milk.

### 3.3. Horizontal Transmission from Breast to Spouse or Vice Versa

While a link between a mother’s HPV DNA type in breast milk and an offspring’s oral HPV type has not been confirmed, a correlation between breast milk HPV type and a spouse’s oral HPV type has been made. A recent study shows that an HPV type (HPV16) detected in breast milk (different from oral or genital HPV type in mother) was identical to spouse’s oral HPV, 6 and 12 months postpartum [38,40]; these results suggest that HPV may be transmitted from the breast to the oral cavity of a spouse or vice versa.

## 4. HPV-Associated HNC (HPV + HNC)

While incidences of alcohol, cigarette, and tobacco-associated HNC have declined (by 50%) in the developed world, due to a decrease in smoking or alcohol consumption, the number of HPV + HNC is increasing [41]. Within the last five decades, the number of OPSSCs due to HPV infections has increased by more than 225% while the numbers of OSCC and LSCC have increased minimally [42,43,44].

**Figure 2:**
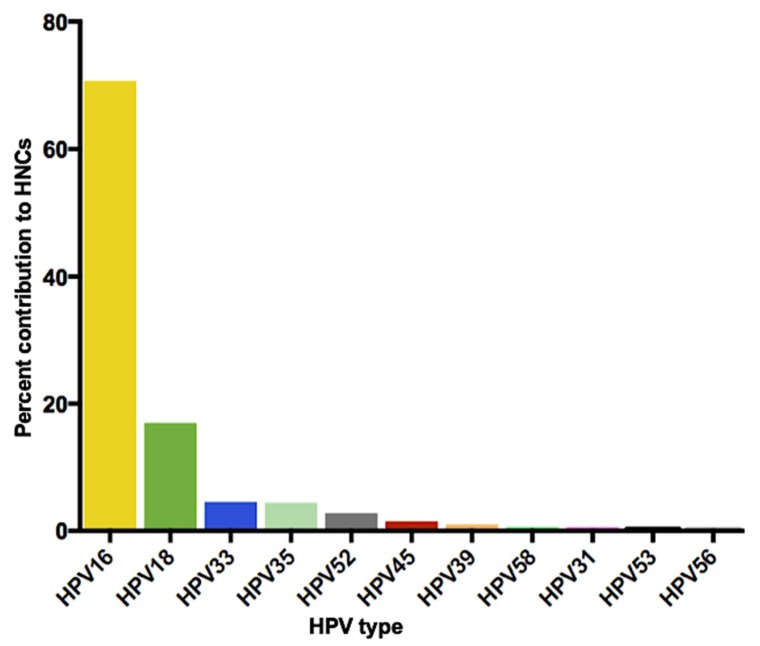
The prevalence/contribution of human papillomavirus (HPV) types to HPV-associated head and neck cancers (HPV+ HNC) (adapted from Table 1).

About 19 high-risk HPV types (16, 18, 26, 31, 33, 35, 39, 45, 51–53, 56, 58, 59, 66, 68, 70, 73, and 82) have been detected in the head and neck regions; they have been detected in oral washes, tonsillar and oropharyngeal wall-swabs, and in laryngeal tissue specimens [44,45]. Although all high-risk HPV types have been detected in the head and neck regions, most head and neck HPV infections (as high as 80%) are cleared, in normal healthy individuals, within 6–20 months of infection [46]; HPV16 has the lowest clearance rate with infection persisting for up to 20 months [46]. Thus, only a percentage of HPV infections, transmitted orally, become persistent infections that would ultimately cause HNC (Table 1 and Figure 2). HPV16 and HPV18 contribute to the majority (~85%) of HPV + HNC cases worldwide while the remaining ~15% of HPV + HNC are caused by HPV33, HPV35, HPV52, HPV45, HPV39, HPV58, etc. (Table 1 and Figure 2) [10,11].

## 5. Influence of Smoking in HPV + HNC

Cigarette smoke has been suggested to contribute to HPV + HNC progression by altering the expression pattern of miRNA in epithelial cells especially miRNA-133a-3p [49,50]; miRNA-133a-3p inhibits proliferation/invasion of HNC [51] and acts as a tumor suppressor miRNA [52]. A recent study demonstrated that smoking or cigarette smoke extract, down-regulates the expression levels of miRNA-133a-3p [50]; down-regulation of miRNA-133a-3p is associated with an increase in the expression levels of epithelial growth factor receptor (EGFR) and Hu-antigen R (involved in post-transcriptional regulation), which contribute to tumor growth [50,53]. Thus, smoking contributes to the progression of HPV + HNC by upregulating the expression of EGFR and HuR. Other mechanisms have been documented or suggested. Studies have shown that smoking increases oral infection with HPV, causes alteration of tonsil cells making them more susceptible to infection, impairs mediators of the immune system, and enhances DNA brakes thus promoting the integration of HPV DNA into the host DNA [12,53,54]. Overall, smoking contributes to the development of HPV + HNC.

## 6. Influence of Immunosuppression in HPV + HNC

Human immunodeficiency virus (HIV) patients especially patients with acquired immunodeficiency syndrome (AIDS) have a compromised immune system (low CD4 counts) and as such, they are 3-times more likely to be infected with HPVs (reviewed in [12]). Although all oncogenic HPV types have been detected in oral washes of patients infected with HIV including AIDS patients [55,56], HIV-infected patients (compared to non-HIV patients) seem to be infected or co-infected more commonly with HPV52, 51, 58, 35, 56, 53, 31, and 59 (reviewed in ref. [12]). Also, HIV/AIDS patients are 3-times more likely to be infected with multiple HPV types compared to non-HIV/AIDS patients [57]; for example, infections with up to 10 different HPV types have been reported in HIV patients (reviewed in ref. [12]). This increases the risk of HPV + HNC in HIV/AIDS patients compared to non-HIV infected population [58]. For instance, in the United States, the incidence of invasive HPV + OPSCC in AIDS patients was 0.0 cases per 100,000 person-years from 1980–1989 (when AIDS was first reported); however, from 1990–1995 and from 1996–2004, HPV + OPSCC incidences increased to 3.9 cases per 100,000 person-years and to 6.5 cases per 100,000 person-years, respectively [59]. In North America as a whole, the incidence of all HPV + HNC in HIV patients (from 1996–2009) increased from 6.8 to 11.4 cases per 100,000 person-years while incidence of non-HPV + HNC in HIV patients decreased (from 41.9 to 29.3 per 100,000 person-years) [60] probably due to a decrease in smoking and/or alcohol consumption. Thus, the number of HPV + HNC cases are increasing in HIV/AIDS population compared to non-HPV + HNC cases (in the same population). It is worth mentioning that in some HIV patients, HPV + HNC (especially HPV + OSCCs) have been reported concurrently with HPV-associated anal cancers [61,62], especially in men who have sex with men. Overall, the number of HPV + HNC cases (i.e., in HIV/AIDS and non-HIV/AIDS populations) are higher in men compared to women [59,63,64].

## 7. Distribution of HPV + HNC between Anatomical Sites

**Figure 3:**
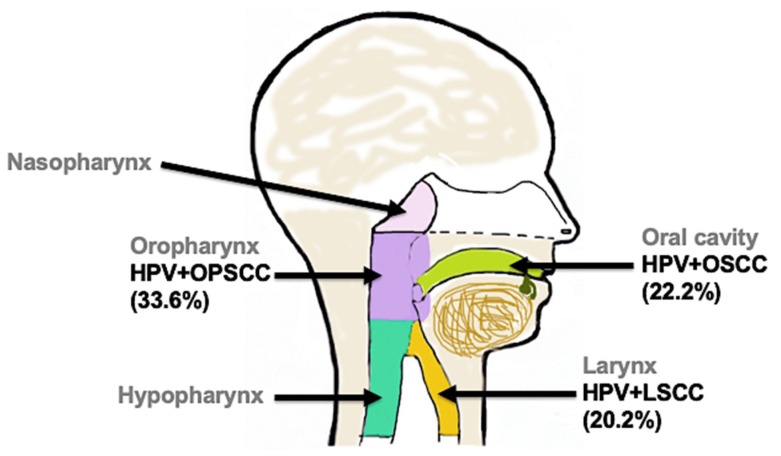
Distribution of human papillomavirus-associated head and neck cancers (HPV + HNC) between anatomical sites. HPV is associated with 33.6% of OPSCC (lavender), 22.2% of OSCC (bright green), and 20.2% of LSCC (gold) worldwide.

As mentioned earlier, oral transmission of HPV and infection of the head and neck region is associated with 25% (average) of HNC cases worldwide; nevertheless, this percent contribution varies between anatomical sites (i.e., between oral, oropharyngeal and laryngeal regions). For instance, as shown in Figure 3, HPVs cause more cases of OPSCC (33.6%) and less cases of OSCC (22.2%) and LSCC (20.2%) worldwide [10,11]. It is unclear why HPV + HNC prevalence varies between anatomical sites and even between some studies; this can be due, but not limited, to the following.

### 7.1. The Source, Quality, and Quantity of Samples that Were Used to Test for HPV Infections or to Diagnose Cases of HPV + HNC between Anatomical Sites

In some studies to assess the contribution of HPV to HNC, oral washes were used, while other studies used tonsillar/oropharyngeal wall swabs/scrapings or formalin-fixed paraffin-embedded oropharyngeal/laryngeal tissues [27,35,44,45,47]. Some of these samples (especially from washes/swabs) may not have had enough cells to test for HPV infection and/or the quality of HPV DNA in the samples may have been poor (if samples were not stored/refrigerated properly); a fewer number of cells in a sample from one anatomical region, compared to another sampled region, may decrease the chances of detecting HPV DNA in samples with less cells. These factors can contribute to the discrepancy in the prevalence of HPV + HNC between anatomical sites.

### 7.2. The Sensitivity of the Assay Used to Detect HPV + HNC

Some HPV DNA detection assays are more sensitive than others. For example, PCR has been shown to be more sensitive than hybridization assays in detecting HPV DNA in buccal swabs; also, nested-PCR has been shown to be more sensitive compared to regular PCR in detecting HPV DNA in oral samples (reviewed in ref. [35]). Thus, if nested-PCR is used to detect HPV DNA in samples from one anatomical region (e.g., oral) while hybridization assays are used to detect DNA in samples from another anatomical region (e.g., oropharyngeal), HPV DNA may be missed in the latter. In summary, the sensitivity of the tests used, in some of these studies, may also be contributing to the discrepancy in the prevalence of HPV + HNC between anatomical sites.

### 7.3. The Type of Biomarker Analyzed and the Anatomical Site of the Sample

Some biomarkers may be more sensitive in detecting HPV + HNC cases at certain anatomical regions but not at other anatomical regions. For example, it has been suggested that the expression level of p16^INK4a^ (a tumor suppressor gene) as well as that of E6/E7 mRNA are more sensitive biomarkers in detecting HPV-associated OPSCC (HPV + OPSCC) than in detecting other cases of HPV + HNC, for example HPV-associated OSCC (HPV + OSCC) and HPV-associated LSCC (HPV + LSCC) [44,65]. Thus, using the expression levels of p16^INK4a^ or E6/E7 mRNA, across the board, as biomarkers to detect all cases of HPV + HNC can lead to the detection of a small number of patients with HPV + OSCC and HPV + LSCC compared to HPV + OPSCC. The low sensitivity of these biomarkers in detecting HPV + OSCC and HPV + LSCC can contribute to the discrepancy in the prevalence of HPV + HNC between the three anatomical regions.

## 8. Distribution of HPV + HNC Cases between Geographical Regions

**Figure 4:**
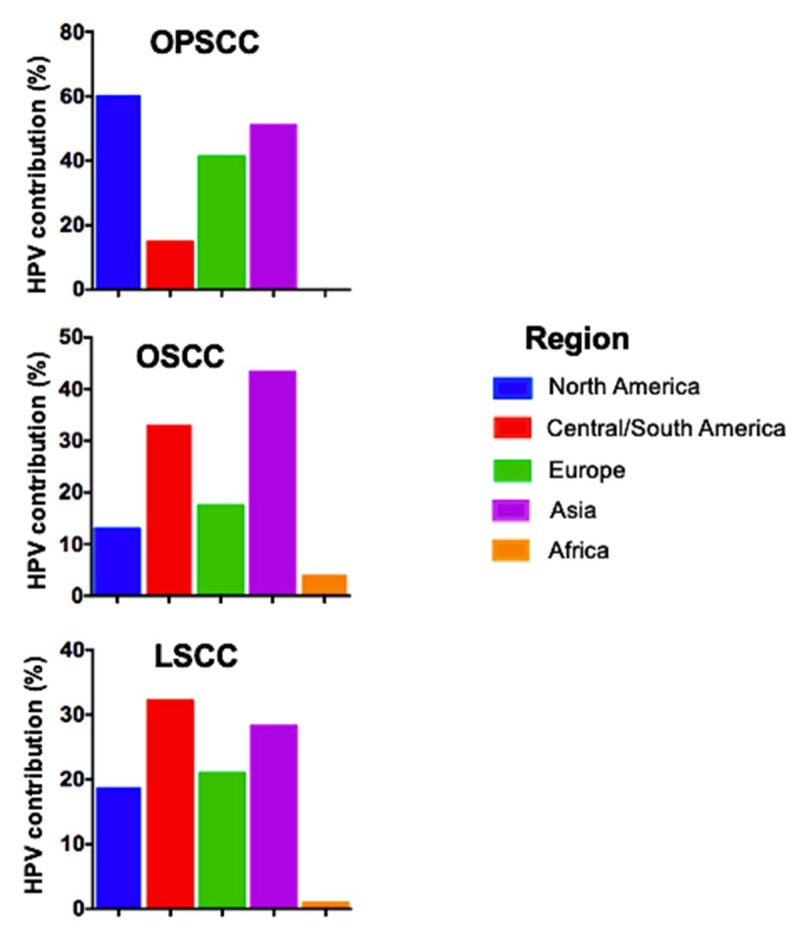
Distribution of human papillomavirus-associated head and neck cancers (HPV + HNC) between geographical regions. Percent HPV + HNC cases in North America, Central/South America, Europe, Asia, and Africa.

Not only does the prevalence of HPV + HNC vary between anatomical regions; it also varies between geographical regions (from one geographical region to another) around the world. HPV + OPSCC is very high in North America compared to Asia, Europe, and Africa (Figure 4). On the other hand, HPV + OSCC is high in Asia compared to Central/South America, Europe, and Africa while HPV + LSSC is high in Central/South America compared to Asia, Europe, and Africa [10,11]. Variation in the prevalence of HPV + HNC between geographical regions may be associated with differences in socio-economic activities such sexual preferences. As mentioned earlier, HPV in the head and neck region is transmitted primarily through orogenital sex and the risk for infection increases with an increase in the number of oral-sex partners. In the Western world (North American and Europe) and in countries with better economy (Central/South American and some parts of Asia), more people have been reported to perform oral sex (84%) [66] compared to people in developing African countries (less than 47% reported) [67,68]. Thus, oral-sex preference in addition to the reasons already discussed above (sensitivity of assay, type of biomarker, source of sample, etc.) may be contributing factors to the discrepancy in the prevalence of HPV + HNC between geographical regions.

## 9. Prevention of HPV Infection and Possibly the Development of HPV + HNC

There are currently three approved prophylactic vaccines (Cervarix, Gardasil-4, and Gardasil-9) to protect against HPV infections; Gardasil-4 has been discontinued in the US. Cervarix is a bivalent vaccine that protects mostly against HPV16 and HPV18 whereas Gardasil-4, a tetravalent vaccine, protects against these HPV types (16 and 18) as well as HPV6 and HPV11. Gardasil-9 is a recent nonavalent vaccine and protects against the 4 HPV types above in addition to HPV31, 33, 45, 52, and 58 (reviewed in ref. [12]). Cervarix is approved for females between 9 and 25 years-old while Gardasil- 4 and Gardasil-9 are approved for both males and females between 9 and 26 years-old [69,70]. Although some of these vaccines have been available for at least a decade, vaccination campaigns have focused mostly on preventing cervical cancer, vaginal cancer, anal cancer, penile cancer and genital warts with little information on the efficacy of the vaccines at preventing HPV + HNC, despite increasing levels of HPV + HNC, especially in men. Recent studies suggest that the vaccines may protect against oral HPV infections. Vaccination with Gardasil-4 or Cervarix vaccines elicited anti-HPV16 and HPV18 IgG antibodies in the oral cavity; albeit three logs lower than serum IgG [71,72]. In another study, it was observed that individuals immunized with Gardasil-4 elicited anti-HPV IgG antibodies in saliva, which neutralized, in vitro, pseudoviruses representing HPV6, 16, and 18, albeit at low titers [73]. The detection of neutralizing anti-HPV antibodies in saliva, following intramuscular immunization, suggests that oral infections with these HPV types and subsequently, a subset of HPV + HNC may be prevented following immunization. In fact, recent studies showed that vaccinated adults compared to the unvaccinated group, had low prevalence (~88% less) of vaccine types (HPV 6, 11, 16 and 18) in their oral cavity [74,75,76,77]; the effect of vaccine on non-vaccine HPV types was not observed in vaccinated and unvaccinated groups. Given these results, it is likely that Gardasil-9 may offer protection, from oral infection, against 9 HPV types (HPV6, 11, 16, 18, 31, 33, 45, 52, and 58).

It worth highlighting that, some of the results described above [71,72] were based solely on antibody titers detected in saliva of vaccine-immunized individuals and/or in vitro neutralization assays using saliva from these individuals. Preclinical studies have gone further to assess, in mice, the potential of Gardasil-4 and other candidate HPV vaccines (under development) to protect against oral HPV infections. In one study, it was demonstrated that mice intramuscularly immunized with Gardasil-4 or with a pan-HPV L2 vaccine (a candidate HPV vaccine targeting broadly neutralizing epitopes on the minor capsid) were protected from oral infection with HPV pseudovirus 16 [78]. Recently, we showed that mice orally immunized with mixed MS2-L2 VLPs (a bacteriophage-based candidate HPV vaccine composed of MS2-16L2/31L2 and MS2-Cons69-86) were protected from infection following oral infection with HPV pseudoviruses 16, 35, 39, 52, and 58 [79]; these HPV types are associated with ~80% of HPV + HNC cases. More importantly, mixed MS2-L2 VLPs candidate vaccine showed complete protection, compared to Gardasil-9 vaccine, from oral infection with HPV35 [79]; HPV35 is the next frequent HPV type (after HPVs16, 18, and 33) associated with HNC (Table 1 and Figure 2). Also, orally immunized mice, with mixed MS2-L2 VLPs, were completely protected from oral infection with HPV pseudovirus 39 whereas Gardasil-9-immunized mice were not protected; Gardasil-9 does not include virus-like particles from HPV35 and HPV39. It is worth mentioning that mixed MS2-L2 VLPs have also been shown to protect against HPV pseudoviruses 11, 16, 31, 33, 45, 53, 56, and 58 at the vaginal region [79,80]; thus mixed MS2-L2 VLPs have the potential to protect against 11 HPV types associated with ~99% of HPV + HNC and 1 HPV type associated with ~32% of recurrent respiratory papillomatosis; estimates are based on the contribution of each HPV type to HPV + HNC from Table 1 and from studies on recurrent respiratory papillomatosis [81,82]. More studies are required to assess the efficacy of mixed MS2-L2 VLPs against HPV6 (associated with ~60% RRP) and to assess the longevity of oral immune responses to mixed MS2-L2 VLPs.

## 10. Treatment of HPV + HNC

The three approved prophylactic vaccines described above are aimed primarily at preventing HPV infections and not to treat HPV infections. Thus, patients who did not receive the vaccines and whose HPV infections persisted and progressed to HPV + HNC have to be treated. HPV + HNC can be treated by surgery, radiotherapy, or chemotherapy. In cases where the cancers have metastasized to other head and neck regions, an adjuvant-based therapy (chemotherapy and radiation), has been used in combination with surgery [83,84]. For non-metastasized cases of HPV + HNC, studies suggest that treatment outcomes (favorable survival, disease-free) are the same regardless of the treatment strategy (either surgery, radiation, or chemotherapy alone) or the order in which a combination treatment was done (i.e., surgery followed by radiation/chemotherapy or vice versa) [83,85,86]. In summary, the treatment outcomes for non-metastasized HPV + HNCs are the same regardless of the treatment options used; HPV + HNC patients have a better prognosis than non-HPV associated HNC patients.

## 11. Challenges Associated with HPV + HNC

Despite advances made in diagnosing cases of HPV + HNC in pre-clinical settings, there are still a lot of challenges in diagnosing HPV + HNC cases in clinical settings and also in treating HPV + HNC patients with HIV/AIDS.

Pap smear screening or HPV DNA testing are recommended or validated methods used in clinical settings to diagnose early stages of cervical cancers. However, there are no recommended or validated methods to screen for early stages of HPV + HNC. Samples from oral washes or swabs can be used to detect HPV in some anatomical regions of the head and neck region; this can provide useful information about HPV infection status of the region and maybe diagnose early stages of HPV + HNC. Nevertheless, there are some limitations associated with this approach; washes/swabs may not have enough cells for HPV analysis. In a situation like this, if a highly-sensitive assay, such as nested-PCR is not used, false-negative results may be reported. Good examples are recent studies by D’Souza et al. [87] and Gipson et al. [88]; HPV-positive DNA were missed in oral rinse samples of HPV + OPSCC patients, who were tested using DNA enzyme-linked immunosorbent assay (DEIA) [87]. The sensitivity of the assay in detecting oral HPV infection was only 51% compared to 81% using serological assay targeting E6 antibodies in serum of the patients. Low sensitivity could have been due to the low number of cells that were in the washes or may be due to poor sensitivity of the DEIA assay in comparison to the serological assay; thus, oral washes/swabs are not approved methods, in clinical settings, to diagnose HPV + HNC. The American Dental Association recently made six clinical recommendations for evaluating oral mucosal lesions (potential malignant disorders) and OSCCs in the oral cavity [89]. Although there was mention of using biopsies (and to some extent, cytology) in evaluating oral lesions, there was no mention of using samples from oral washes/swabs to diagnose HPV + HNC.

Serological tests targeting E2, E6, and E7 antibodies can be used as an early diagnostic test for HPV + HNC especially HPV + OPSCC [90,91]. However, not all HPV + HNC patients are seropositive for E6 and/or E7 antibodies [92]; additionally, not all patients who are seropositive for some of these antibodies will develop HPV + HNC. This makes reliance on seropositivity, as a pre-diagnostic marker for HPV + HNC, very challenging. Overall, diagnosis of HPV + HNC lags behind, making it very challenging to implement early preventive interventions.

The last but not the least challenge associated with HPV + HNC is the indirect effect of highly active anti-retroviral treatment (HAART) on HPV-associated cancers. While most patients with HIV/HPV co-infections on HAART therapy have a reduced risk of developing HPV-associated cancer (cervical cancer) compared to patients on non-HAART therapy (reviewed in ref. [93]), some HIV/HPV patients on HAART therapy seem to have a high risk of developing HPV-associated oral lesions/cancers [62,94,95]; the risk is suggested to be due to longer life expectancy as a result of HAART therapy. It is unclear why HAART therapy may have different indirect effects on HPV + HNC versus HPV-associated cervical cancers. Does HAART therapy increase the life expectancy of HIV patients with HPV oral lesions and not those with cervical cancer? Future research needs to be conducted to address this discrepancy.

## 12. Prospects for the Future

In the long-run, studies/measures have to be taken to:

### 12.1. Reduce Discrepancy in the Prevalence of HPV + HNC Cases between Studies, Anatomical Sites, and/or between Geographical Regions

This can be achieved in a number of ways as described below.

The same or approximate number of cells in samples from the three anatomical regions of the head and neck should be used in assays in order to avoid false negative results in some cases. Thus, it is advisable to collect and confirm that all samples from the three anatomical regions, especially from oral washes/scrapings, have enough cells before moving forward with analysis. A hundred thousand cells from three mucosal scrapings have been shown to increase HPV detection (reviewed in ref. [35]). Thus, using the same number of cells (may be ~100,000), from each of the three different anatomical regions of the head, may reduce the discrepancy in the number of HPV + HNC cases between anatomical sites. This cannot be achieved without using the same type(s) of assays/markers, across the board, to assess HPV status in HNC.

Assays that target HPV nucleic acids and are very sensitive (for example, assays that utilize nucleic acid amplification; nested-PCR, real-time PCR, etc.) should be used across the board when assessing the association of HPV with all cases of HNC (OSSC, OPSCC, and LSCC); many of these assays can be done using commercially available kits [89]. If HPV nucleic acid is amplified using these assays, DNA hybridization assays using cells from suspected lesions can then be used to further validate the results; the nuclei of HPV + HNC cells but not HPV-negative HNC cells should hybridize with probes. On the other hand, if the expression levels of p16^INK4a^ protein and/or E6/E7 mRNA have to be used to assess the association of HPV with HNC cases, caution should be taken because p16^INK4a^ and E6/E7 mRNA are sensitive biomarkers for HPV + OPSCC but not for HPV + OSCC or HPV + LSCC. Moreover, it should be noted that high levels of p16^INK4a^ protein may not necessarily be due to HPV; it could be due to other factors: inflammation, cell ageing, or other microbial infections [96,97,98]. Thus, using these biomarkers to compare the prevalence of HPV + HNC between these three anatomical sites can be misleading.

Accurately pinpointing/reporting the anatomical region(s) where samples are taken to assess cases of HPV + HNC may minimize discrepancy in the prevalence of HPV + HNC between anatomical regions. The oral region, the pharynx and the larynx (Figure 3) are all connected and their anatomy is very complex [99]; this makes it very challenging to know specifically from which anatomical region samples are collected to assess HPV status. Oral washes, oropharyngeal wall-swabs, and even biopsies from in-between these regions have the potential to contain cells from both regions. Thus, tissue-specific test or anatomic region-specific test, using specific markers (if available), should be done on samples to assess the percentage of oral cells versus oropharyngeal cells prior to testing for HPV status. This will help shed additional light on the anatomical origin of cells in question.

### 12.2. Assess whether HPV Can Be Transmitted through Other Means such as Fomites or Breast Milk

There is a need to assess whether reusable fomites such as intra-oral transducers or oral probes used in intra-oral ultrasonography can horizontally transmit HPV to the oral/pharyngeal regions. HPV is a non-enveloped virus and thus, the virus or its DNA may persist for a while in fomites. For example, HPV DNA has been detected (by PCR) in transvaginal ultrasound probes and colposcope even after recommended low-level disinfection with quaternary ammonium compound and chlorhexidine-wipes [100,101]. The detection of viral DNA does not imply that any of the tested fomites had infectious HPV; however, given the fact that *Staphylococcus aureus* was cultured from samples taken from these probes (transvaginal ultrasound and colposcope), studies are needed to assess whether intra-oral transducers/sensors or oral probes carry HPV DNA and infectious HPV.

More studies are also needed to assess whether HPV can be transmitted from the oral cavity to the breast (or vice versa) and whether HPV has a role in some cases of breast cancers. While a solid link between HPV and HNC (including anogenital cancers) has been made, a link between HPV and some cases of breast cancers is still debatable/controversial. As mentioned earlier, HPV16 has been detected in the breast milk from a mother and in oral samples from her spouse [38,40]; furthermore, an increasing number of studies have also detected HPV DNA and the expression of E7 protein in breast cancer samples in Europe [102,103], North America [104], Central/South America [105,106], and Asia/Oceania [107,108,109]. Thus, there is increasing data and it is likely that HPV may be associated with some types of breast cancers. There is a potential route/means through which HPV can be transmitted to the breast and there are cells in the breast that may have the potential to be permissive to HPV infection. For example, during intimate contact, oral fluids come in contact with nipples, which contain openings that are linked to breast milk ducts. The milk ducts are lined with specialized epithelial cells and HPV normally infects epithelial cells especially those with secretory functions. Given this link (possibility of a transmission route and the presence of target cells), there is an urgent need to assess whether HPV can establish persistent infection in breast epithelial cells lining the milk duck or the breast and whether the virus is associated with some cases of breast cancers.

### 12.3. Assess whether HPV Can Be Transmitted Parenterally or through Blood Transfussion

As mentioned above, HPVs associated with head and neck cancers including anogenital cancers/warts are believed to be transmitted primarily through sexual contacts, where they establish localized infection. Nevertheless, HPV DNA has been detected in blood [110], gastrointestinal cancers [111], colorectal cancer [112], lung cancer [113], etc. prompting the question whether HPV DNA in these organs may be as a result of parenteral transmission/blood transfusion. A recent preclinical study suggests that papillomaviruses can be transmitted through blood and can establish infections in the infected animal [114]. Rabbits or mice intravenously infected with cottontail rabbit papillomavirus (CRPV) or mouse papillomavirus (MmuPV1), respectively, showed viral replication (with infections in the stomach) and the rabbits developed tumors at the skin and mucosal sites; moreover, naïve animals transfused with blood from infected animals were also infected [114]. Taken together, these preclinical data with CRPV and MmuPV1 suggest that HPV may also be transmitted through the parenteral route/blood transfusion. In fact, a recent study has detected HPV (16, 18, 32, 33, 45, etc.) DNA in peripheral blood mononuclear cells of ~ 6.5% asymptomatic blood donors [115]. Thus, studies are urgently needed to assess whether HPV has the potential to be transmitted parenterally or through blood transfusion given the fact that transfused blood is not screened for HPV infection unlike other infectious agents such as HIV, hepatitis B and C viruses, human T-cell lymphotropic virus, West Nile virus, Zika virus, *Treponema pallidum*, etc. [116].

## 13. Conclusions

In summary, HPV at the head and neck region is transmitted orally with oral sex contributing to the majority of head and neck-associated HPV transmissions/infections. While progress (in terms of treatment) has been made within the last few decades to increase overall survival of HPV + HNC patients, screening and diagnosis of HPV + HNC lags behind cervical cancer. Future screening techniques should focus on using HPV DNA as a marker in diagnosing cases of HPV + HNC regardless of anatomical region; in suspected cases of HPV + OPSCC, the expression levels of p16^INK4a^ protein in the oropharyngeal cells as well as seropositivity to E6/E7 antibodies in serum should be assessed. E6 antibodies have been detected in serum, more than 10 years, prior to the diagnosis of HPV + OPSCC; seropositivity is a sensitive method to diagnose HPV + OPSCC [91,117].

Limited studies suggest that current HPV vaccines have the potential to protect against oral HPV infections. More studies are needed to assess the efficacy of current and candidate HPV vaccines against oral HPV infections and ultimately HPV + HNC (especially in HIV patients). In addition to these, therapeutic vaccines against HPV + HNC are needed. Preclinical studies have shown that therapeutic HPV vaccines targeting E6 and E7 oncogenes have the potential to reduce tumor size and increase mice survival rate [118,119]. More recently, clinical trials have shown that some of these vaccines can reduce cervical intraepithelial neoplasia (CIN)3 to CIN2 or to low-grade squamous intraepithelial lesions in some women; in some cases, patients were tumor free after 21 months (reviewed in ref. [120]). Studies are required to assess the efficacy of these candidate therapeutic HPV vaccines against HPV + HNC.

## Figures and Tables

**Table 1:** The contribution of each human papillomavirus (HPV) type to head and neck cancer (HNC) cases worldwide.

HPV Type	Worldwide Contribution to HNCs *
HPV16	70.7%
HPV18	14%–17%
HPV33	4.5%
HPV35	4.5%
HPV52	2.7%
HPV45	1.5%
HPV39	1.04%
HPV58	0.6%
HPV31	0.56%
HPV53	0.3%
HPV56	0.25%

* Data summarized from refs. [10,11,47,48].
